# Towards automatic classification of cardiovascular magnetic resonance Task Force Criteria for diagnosis of arrhythmogenic right ventricular cardiomyopathy

**DOI:** 10.1007/s00392-022-02088-x

**Published:** 2022-09-06

**Authors:** Mimount Bourfiss, Jörg Sander, Bob D. de Vos, Anneline S. J. M. te Riele, Folkert W. Asselbergs, Ivana Išgum, Birgitta K. Velthuis

**Affiliations:** 1 Department of Medicine, Division of Cardiology, University Medical Center Utrecht, Utrecht University, Heidelberglaan 100, 3584 CX Utrecht, The Netherlands; 2 Department of Biomedical Engineering and Physics, Amsterdam University Medical Centers, University of Amsterdam, Amsterdam, The Netherlands; 3 Amsterdam Cardiovascular Sciences, Heart Failure & Arrhythmias, Amsterdam, The Netherlands; 4 Netherlands Heart Institute, Utrecht, The Netherlands; 5 Institute of Cardiovascular Science, Faculty of Population Health Sciences, University College London, London, UK; 6 Health Data Research UK and Institute of Health Informatics, University College London, London, UK; 7 Department of Radiology and Nuclear Medicine, Amsterdam University Medical Centers, University of Amsterdam, Amsterdam, The Netherlands; 8 Department of Radiology, University Medical Center Utrecht, Utrecht University, Utrecht, The Netherlands

**Keywords:** Arrhythmogenic right ventricular cardiomyopathy, Cardiac magnetic resonance imaging, Deep learning, Automatic segmentation

## Abstract

**Background:**

Arrhythmogenic right ventricular cardiomyopathy (ARVC) is diagnosed according to the Task Force Criteria (TFC) in which cardiovascular magnetic resonance (CMR) imaging plays an important role. Our study aims to apply an automatic deep learning-based segmentation for right and left ventricular CMR assessment and evaluate this approach for classification of the CMR TFC.

**Methods:**

We included 227 subjects suspected of ARVC who underwent CMR. Subjects were classified into (1) ARVC patients fulfilling TFC; (2) at-risk family members; and (3) controls. To perform automatic segmentation, a Bayesian Dilated Residual Neural Network was trained and tested. Performance of automatic versus manual segmentation was assessed using Dice-coefficient and Hausdorff distance. Since automatic segmentation is most challenging in basal slices, manual correction of the automatic segmentation in the most basal slice was simulated (automatic^−basal^). CMR TFC calculated using manual and automatic^−basal^ segmentation were compared using Cohen’s Kappa (κ).

**Results:**

Automatic segmentation was trained on CMRs of 70 subjects (39.6 ± 18.1 years, 47% female) and tested on 157 subjects (36.9 ± 17.6 years, 59% female). Dice-coefficient and Hausdorff distance showed good agreement between manual and automatic segmentations (≥ 0.89 and ≤ 10.6 mm, respectively) which further improved after simulated correction of the most basal slice (≥ 0.92 and ≤ 9.2 mm, *p* < 0.001). Pearson correlation of volumetric and functional CMR measurements was good to excellent (automatic (*r* = 0.78–0.99, *p* < 0.001) and automatic^−basal^ (*r* = 0.88–0.99, *p* < 0.001) measurements). CMR TFC classification using automatic^−basal^ segmentations was comparable to manual segmentations (κ 0.98 ± 0.02) with comparable diagnostic performance.

**Conclusions:**

Combining automatic segmentation of CMRs with correction of the most basal slice results in accurate CMR TFC classification of subjects suspected of ARVC.

**Graphical abstract:**

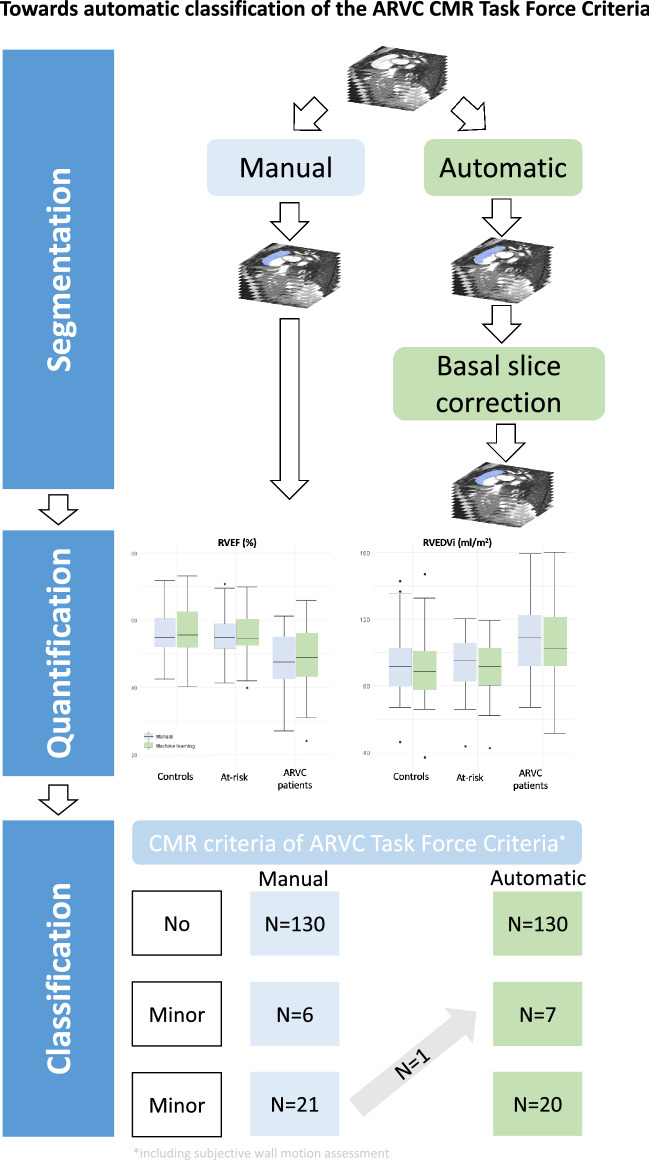

**Supplementary Information:**

The online version contains supplementary material available at 10.1007/s00392-022-02088-x.

## Background

Arrhythmogenic right ventricular cardiomyopathy (ARVC) is an inherited heart disease that is characterized by ventricular dysfunction, predominantly affecting the right ventricle (RV), and potentially life-threatening ventricular arrhythmias. Accurate recognition of this disease is vital since the implantation of an implantable cardioverter defibrillator can be life-saving. ARVC is diagnosed according to the revised 2010 Task Force Criteria (TFC) [1]. Apart from electrical and family history criteria, an important role is given to the assessment of ventricular dysfunction and structural alterations. Cardiac magnetic resonance (CMR) imaging is the modality of choice for the assessment of cardiac function and dimensions in ARVC [2] since the asymmetric geometry and the position of the RV in the chest hampers visualization of the entire RV by 2D echocardiography [3].

The CMR TFC are based on RV regional wall motion abnormalities combined with cut-off values for RV ejection fraction (EF) or sex-specific cut-off values for RV indexed end-diastolic volume (EDVI)[1]. CMR can deliver one minor or two major points of the necessary four TFC points for an ARVC diagnosis. Therefore, accurate RV assessment is essential. Segmenting CMRs to measure functional and structural parameters is a laborious task, taking about 25 min to segment both ventricles in end-diastole (ED) and end-systole (ES) [4, 5]. Notably, RV segmentation takes two-thirds of this segmentation time and is prone to intra- and inter-observer variability [6]. RV segmentation difficulties can arise from the trabeculated and complex RV geometry [7, 8]. In ARVC, RV and left ventricular (LV) anatomy can be further complicated by pathological wall thinning and aneurysms due to fibrofatty replacement of the myocardial wall [2]. As a consequence, CMR misinterpretations are a key cause of over-diagnosis in ARVC [2]. The use of automatic methods for the segmentation of the ventricles may overcome these challenges. Over the last few years many state-of-the-art deep learning segmentation approaches for short-axis CMR have been developed [4, 9–11]. For automatic LV segmentation such methods can achieve performance level of human experts [12, 13]. However, previous studies also demonstrated that in manual and automatic segmentation of short-axis CMR, the largest disagreements and errors occur in the most basal and apical slices [8, 12–15]. Moreover, previous methods have often been evaluated on CMR datasets with limited pathology especially related to the RV. In contrast, this study included a large hospital population being evaluated for ARVC, including subjects with structurally normal hearts and those with complex structural abnormalities. In this work we apply a previously validated state-of-the-art segmentation approach [16] on a large heterogeneous hospital population of patients suspected of ARVC. The purpose of this study was to (i) evaluate our previously developed deep learning segmentation approach for RV and LV CMR assessment in patients suspected of ARVC and (ii) evaluate the clinical implication of this approach for classification of the CMR TFC in subjects suspected of ARVC.

## Methods

### Study population

We included a consecutive cohort of subjects suspected of ARVC who underwent CMR as part of their clinical evaluation between 2014 and 2019 at the University Medical Center (UMC) Utrecht. This yielded 241 subjects, of whom 14 were excluded because of an equivocal diagnosis (ARVC neither confirmed nor rejected) (*n* = 12), prior chemotherapy (*n* = 1) and imaging artefacts due to irregular heart rhythm (*n* = 1). This led to a study population of 227 subjects who were classified into three groups: (1) ARVC patients diagnosed according to the 2010 TFC (*n* = 53); (2) family members at-risk of developing ARVC (*n* = 96); and (3) control subjects initially suspected of ARVC but in whom ARVC was excluded after full clinical assessment (*n* = 78). Diagnosis in the control patients included RV outflow tract tachycardia (*n* = 45), premature ventricular contractions/non-sustained ventricular tachycardia (*n* = 19), mutation-negative family members of mutation-positive ARVC patients (*n* = 3), healthy athletes (*n* = 3), syncope without a cardiac cause (*n* = 3) repolarization abnormalities with a structurally normal heart (*n* = 3) and pectus excavatum (*n* = 2). This study was reviewed by the UMC Utrecht Institutional Review Board and was granted a waiver of informed consent.

### ARVC diagnosis

ARVC diagnosis was based on the revised 2010 diagnostic TFC [1]. In short, these consensus-based criteria rely on major and minor criteria for six different categories: (1) global and regional dysfunction and structural alterations; (2) tissue characterization; (3) repolarization abnormalities; (4) depolarization/conduction abnormalities; (5) arrhythmias; and (6) family history/genetics. In each of these six categories subjects can score a minor criterium (one point), a major criterium (two points) or no criteria (0 points). A definite ARVC diagnosis was made if a subject has at least four points. The first category can be assessed by CMR, with minor criteria for regional RV wall motion abnormalities plus RVEF > 40 to ≤ 45% or RVEDVI ≥ 100 to < 110 ml/m^2^ (males) or ≥ 90 to < 100 mL/m^2^ (females) and major criteria for RV regional wall motion abnormalities plus RVEF ≤ 40% or RVEDVI ≥ 110 ml/m^2^ (males) or ≥ 100 ml/m^2^ (females) [1].

### CMR dataset

All subjects underwent CMR using either 1.5 or 3 Tesla Ingenia or Achieva Philips scanners (Best, the Netherlands). The CMR dataset consisted of conventional steady-state free precession sequence short-axis and longitudinal-axis (4-chamber, 2-chamber and 3-chamber of both ventricles) cine CMR images acquired during breath holds. For this work, we only included the short-axis CMR volumes consisting of 12–18 contiguous slices covering both ventricles. The short-axis imaging parameters were as follows: each slice containing 25 to 40 phases covering one cardiac cycle with repetition time 2.6–3.4 ms and echo time 1.3–1.7 ms, flip angle 45–60 degrees. The CMR images have an in-plane resolution ranging from 1.11 to 1.45 with a slice thickness varying from 7 to 10 mm. Furthermore, reconstruction matrix of images ranges from 240 × 240 to 288 × 288 voxels. Expert radiology technicians made manual reference segmentations of the RV and LV endocardium for all CMR slices at ED and ES time frames. Both time points were manually chosen by the same experts. The CMR segmentation protocol was published previously [17] and adheres to the guidelines of the Society of Cardiovascular Magnetic Resonance (SCMR) [18]. Furthermore, the presence of RV and/or LV wall motion abnormalities was visually evaluated by an experienced cardiovascular radiologist on all available cine images and used for the calculation of the CMR TFC.

### Automatic segmentation of CMR

Prior to segmentation, voxel intensities in CMR scans were normalized by rescaling the values between [0,1] based on their 1^st^ and 99^th^ percentiles per scan. Furthermore, voxels intensities below or above the 1^st^ and 99^th^ percentiles were clamped to 0 and 1, respectively.

To perform automatic segmentation of RV and LV in the 2D short-axis CMR images, we trained a Bayesian Dilated Residual Neural Network (DRN) [19] that was previously developed and evaluated by Sander et al*. *[16]. The Bayesian DRN was based on the original DRN from Yu et al*. *[19] for image segmentation. To convert the original DRN [19] into a Bayesian DRN, we implemented *Monte Carlo* dropout (MC dropout) introduced by Gal & Ghahramani [20]. Using a Bayesian, i.e. MC dropout approach is advantageous because multiple predictions for the same voxel can be averaged to obtain an improved final prediction per voxel [16]. Furthermore, architecture and parameters of the Bayesian DRN were identical to the model described in [16]. The network used a 2D CMR image as input and had three output channels, each providing probability for the LV, RV or background. Softmax probabilities were calculated over the three tissue classes. To train the model a combination of soft-Dice [21] and cross-entropy was used as loss function. For completeness, we provide the equations for both loss functions:

$$\text{soft-Dice}_\text{c}= \frac{\sum_{i=1}^{N} {{R}_{c}(i) A}_{c}(i)}{{\sum }_{i=1}^{N}{R}_{c}(i) +{\sum }_{i=1}^{N}{A}_{c}(i) } \, ,$$

where *N* denotes the number of voxels in an image, ${R}_{c}$ is the binary reference image for class *c* and ${A}_{c}$ is the probability map for class *c*.

$$\text{Cross-Entropy}_{\text{c}}= - \sum_{i=1}^{N}{t}_{ic}\mathrm{log}\,p\left({y}_{i}=c |{x}_{i}\right),$$

where $p$ denotes the probability for a specific voxel ${x}_{i}$ with corresponding reference label ${y}_{i}$ for class $c$; and ${t}_{ic}=1$ if ${y}_{i}=c$; and 0 otherwise. Hyper-parameters of the network were determined in our previous work [16] using CMR images from the MICCAI 2017 Automated Cardiac Diagnosis Challenge (ACDC) [12]. Therefore, no validation set was required in the current work.

To train the model, patches of 160 × 160 voxels were randomly chosen from the training set. Training data were augmented by 90 degree rotations, elastic deformations and gamma transformations of the images. The model was trained for 160,000 iterations using mini-batch stochastic gradient descent with batch-size 16 and Adam as optimizer [22]. Learning rate was set to 0.001 and decayed with a factor of 0.1 after every 40,000 steps. To increase generalization performance weight decay was used and set to 0.0005. Furthermore, dropout percentage was set to 0.1. Enabling MC dropout during testing, tissue class per voxel was determined using the mean softmax probabilities over 15 samples. Voxel wise segmentation may result in isolated (small clusters of) voxels. To address this, only the largest 3D connected component for each class was retained in the automatic segmentations.

### Simulation of the correction of automatic segmentation

Previous research demonstrated that most segmentation inaccuracies occur in the most basal slice on the CMR [8, 12–15]. To evaluate whether these inaccuracies of our method impact TFC classification, correction of the automatic segmentation in the most basal slice of each CMR volume was simulated. This was achieved by replacing the automatic segmentation of the most basal slice with the corresponding manual reference defined by specially trained radiology technicians as a part of a regular clinical workup. We refer to this scenario as *automatic*^*−basal*^ hereafter.

### Automatic ED/ES phase selection

Accurate identification of ED and ES phase in the cardiac cycle is a prerequisite to automatically compute RVEDV and RVESV. To show the potential of the method to automatically determine the ED and ES phase, we automatically segmented all CMR volumes of the patients in the test set, and derived the RV and LV volumes for all time points of the cardiac cycle. For each patient ED was identified as the phase in which the fully automatically segmented volume was maximal and ES as the phase in which the volume was minimal. Automatically identified phases were compared with the manually selected phases using Bland–Altman analysis. In these plots (e.g. Figure 2a), the distance between automatically and manually selected phases is expressed as percentage of a complete cardiac cycle. Evaluation was performed for RV and LV separately, and for automatic and automatic^−basal^ segmentations separately.

### Evaluation of automatic segmentation

To evaluate performance of the automatic segmentation method 3D Dice-coefficient and 3D Hausdorff distance between manual and automatic segmentations were computed. For this, the 2D automatic segmentation masks were stacked into a 3D volume per patient and cardiac phase. The Dice-coefficient quantifies overlap between manual and automatic segmentation and its value ranges between 0 and 1. A higher Dice-coefficient indicates better agreement between manual and automatic segmentations. The Hausdorff distance evaluates segmentation along the boundary of the target structure by measuring the maximum distance between manual and automatic segmentation. Qualitative performance of the automatic segmentation method was visually assessed. To investigate whether segmentation errors accumulate at specific slice locations in the CMR volume the distribution of segmentation errors over slice location was computed. For this, four slice locations in a volume were distinguished: (i) most apical slice; (ii) most basal slice; (iii) mid-ventricular slices and (vi) slices located below the apex or above the base of the heart. Furthermore, to evaluate the clinical implications of our automatic CMR segmentation approach for the classification of the CMR TFC in subjects suspected of ARVC, the following CMR measurements were computed for manual, automatic and *automatic*^*−basal*^ segmentations: LV end-diastolic volume (EDV); LV end-systolic volume (ESV); LV stroke volume (SV); LVEF; RVEDV; RVESV; RVSV and RVEF.

### Statistical analysis

Statistical analysis was performed using RStudio Version 1.3.1093 (Boston, MA, USA) and IBM SPSS Statistics (version 25, USA). Continuous values were presented as mean ± standard deviation or median [interquartile range]. Categorical data were displayed as absolute frequency (*n*) and percentages (%). For continuous comparisons of two groups, two-tailed Student’s *t* test was used. For continuous comparisons of three or more groups, one-way ANOVA was used. Categorical data were compared using the chi-squared test. A *p* value of < 0.05 was considered significant. Comparison of automatic and manual absolute CMR measurements were assessed using Bland–Altman analysis and the Pearson correlation coefficient (*r*). CMR TFC was first classified using visual assessment of wall motion abnormalities and manually derived RVEDVI and RVEF, and next using visual assessment of wall motion abnormalities and automatically derived RVEDVI and RVEF.

CMR TFC classification agreement between manually vs. automatically derived CMR measurements was assessed using Cohen’s kappa (κ). Furthermore, sensitivity and specificity of CMR TFC by manual and automatic approach was determined and compared using the McNemar test.

## Results

### Study population

We included 70 subjects in the training set (mean age 39.6 ± 18.1 years, 47% female) and 157 subjects in the test set (mean age 36.9 ± 17.6 years, 59% female). Patient characteristics are shown in Table 1. The test set included 37 ARVC patients, 66 at-risk family members and 54 controls subjects. The distribution of subjects across the three patient categories was the same for training and test sets (34% controls, 42% at risk, 24% ARVC patients). No statistically significant difference in sex existed between the three subgroups (*p* = 0.37), but at-risk family members were younger than ARVC patients (*p* = 0.021) and controls (*p* < 0.001). ARVC patients had a median of 5 [4–6] diagnostic TFC points, while at-risk family members had a median of 2 [1–3] points (*p* < 0.001). In total, 84% of ARVC patients and 3% of at-risk family members had minor or major CMR TFC (RV wall motion abnormalities combined with abnormal RVEF or RVEDVI cut-off values). Among 103 ARVC patients and at-risk family members, 90 (87%) carried a pathogenic variant, mostly in plakophilin-2 (n = 57, 63%) followed by phospholamban (*n* = 26, 29%) and desmoplakin (n = 5, 6%).

**Table 1:** Baseline characteristics

	ARVC patients (*n* = 37)	At-risk ARVC group (*n* = 66)	Control group (*n* = 54)	*p* value
Demographics				
Age at CMR (years)	39.1 ± 19.0	30.7 ± 16.2^b,c^	42.9 ± 15.9	< 0.001
Female (%)	20 (54)	43 (65)	29 (54)	0.37
Proband (%)	10 (27)	0 (0)^c^	na	< 0.001
Genetic status				
Pathogenic variant	36 (97)	56 (85)	na	0.06
PKP2 (%)	24 (71)	33 (59)		
PLN (%)	4 (12)	22 (39)		
DSP (%)	4 (12)	1 (2)		
Other (%)	4 (12)	0		
Clinical phenotype				
Total TFC score	5 [4–6]^a^	2 [1–3]^b,c^	0	< 0.001
Repolarization criteria				
Minor	10 (27)	0 (0)		
Major	8 (22)	3 (5)		
Depolarization criteria				
Minor	23 (62)	9 (14)		
Major	0 (0)	0 (0)		
Arrhythmia criteria				
Minor	25 (68)	6 (9)		
Major	2 (5)	0 (0)		
Structural criteria				
Minor	6 (16)	1 (3)		
Major	25 (68)	0 (0)		

*ARVC* arrhythmogenic right ventricular cardiomyopathy, *CMR* cardiac magnetic resonance imaging, *DSP desmoplakin*, *PKP2 plakophilin-2*, *PLN phospholamban*, *TFC* Task Force Criteria

^a^Significant difference between control and ARVC patients

^b^Significant difference between control and at-risk subjects

^c^Significant difference between ARVC patients and at-risk subjects

### Assessment of segmentation performance

**Fig. 1:**
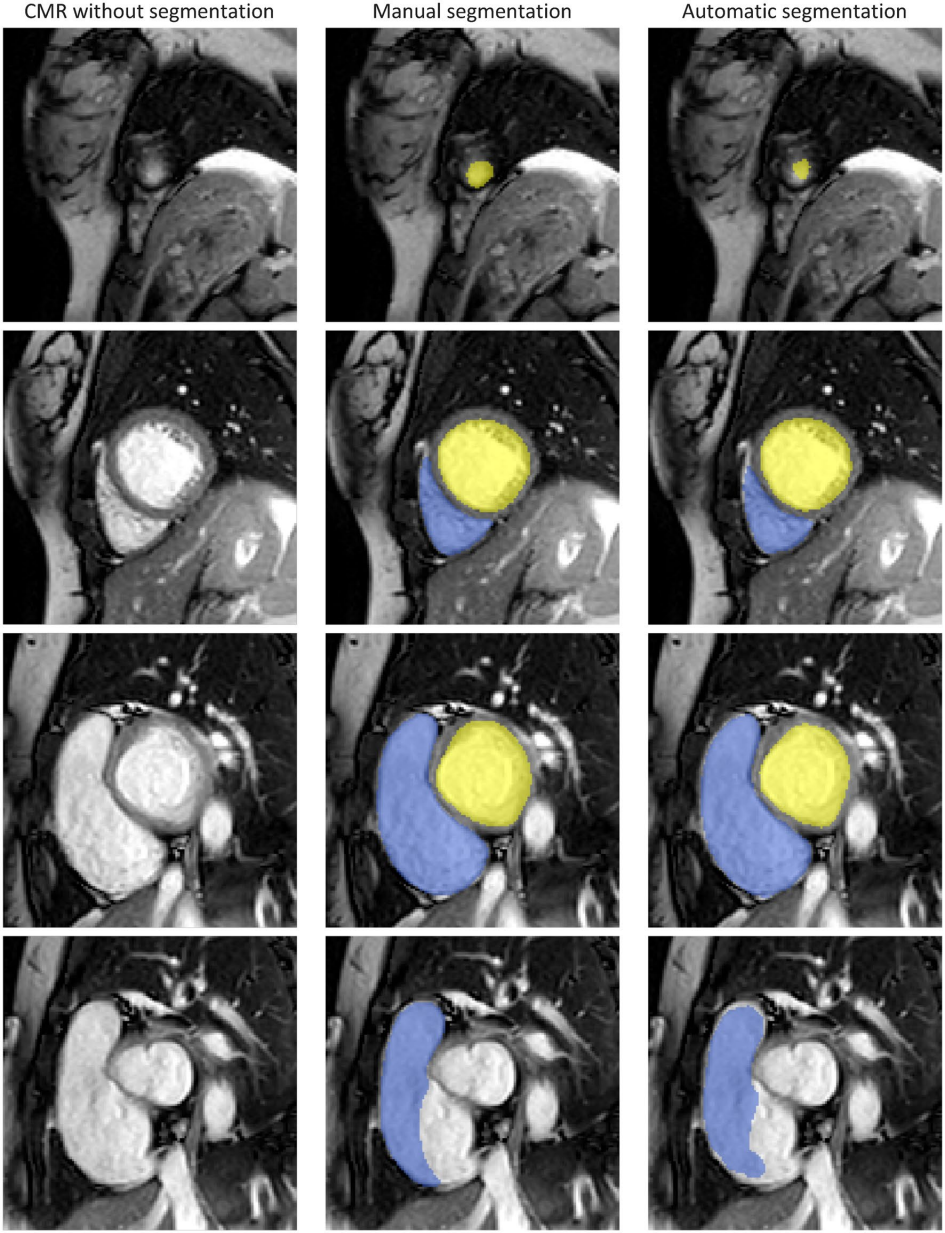
example automatic segmentation vs. manual segmentation. Qualitative segmentation results for left (yellow) and right (blue) ventricles at end-systole for a patient included in the test set. Columns depict raw CMR (first column), CMR with manual reference segmentation (second column) and CMR with automatic segmentation (third column). Rows show apical, mid-ventricular and most basal slices for LV (third row) and RV (fourth row), respectively

Table 2 lists quantitative results of the automatic segmentation. The automatic method achieved mean Dice-coefficient for ED and ES 0.96 ± 0.01 and 0.93 ± 0.03, respectively, for the LV and 0.93 ± 0.04 and 0.89 ± 0.04, respectively, for the RV. Visual assessment of automatic segmentation results depicted in Fig. 1 reveal that performance was higher for mid-ventricular slices (second and third rows Fig. 1) compared with apical and basal slices (first and fourth row Fig. 1), while an under-segmentation of trabeculated areas occurred in the apical slices (first row Fig. 1). Furthermore, as depicted in Supplementary Fig. 1, visual assessment of the manual reference segmentation revealed a high variability of the RV shape in the basal slices in both ED and ES time points. Furthermore, as listed in Table 3, comparison of automatic with manual reference segmentations disclosed that on average 24.5% of the segmentation errors, i.e. misclassified voxels were located in the most basal slice (30.7 and 18.3% for RV and LV, respectively). In contrast, on average only 6.5% of the errors were located in an apical slice (5.4 and 7.6% for RV and LV, respectively).

**Table 2:** Segmentation performance of deep learning segmentation model

	End-diastole		End-systole	
	LV	RV	LV	RV
Dice-coefficient				
Automatic	0.96 ± 0.01	0.93 ± 0.03	0.93 ± 0.04	0.89 ± 0.04
Automatic + correction	0.97 ± 0.01	0.95 ± 0.02	0.95 ± 0.02	0.92 ± 0.03
Hausdorff distance				
Automatic	6.42 ± 2.26	10.42 ± 2.99	6.58 ± 2.73	10.60 ± 3.50
Automatic + correction	5.07 ± 2.27	9.19 ± 3.19	5.52 ± 2.47	9.09 ± 3.05

Segmentation performance of deep learning segmentation model in terms of Dice-coefficient (higher is better) and Hausdorff distance (in millimeter, lower is better). *Automatic* + *correction* refers to the scenario in which the most basal slice of each automatic segmentation volume was replaced with the corresponding manual reference. Depicted values specify mean ± standard deviation

*LV* left ventricle, RV right ventricle

**Table 3:** Distribution of segmentation errors across slices

	Basal (%)	Mid-ventricular (%)	Apical (%)	Slices above/below base/apex (%)
LV	18.3	70	7.5	4.2
RV	30.7	61	5.4	2.9

Percentage of segmentation errors per target structure (LV and RV) located in basal, apical, all mid-ventricular or slices above base and below apex

*LV* left ventricle, RV right ventricle

Table 2 lists segmentation results after the simulated correction of the automatic RV and LV segmentation in the most basal slice. The results show an increased segmentation performance: mean Dice-coefficient for the ED and ES are 0.97 ± 0.01 and 0.95 ± 0.03 (vs. 0.96 ± 0.01 and 0.93 ± 0.03 uncorrected), respectively for the LV and 0.95 ± 0.02 and 0.92 ± 0.03 (vs. 0.93 ± 0.04 and 0.89 ± 0.04 uncorrected), respectively for the RV (*p* < 0.001 [one side Wilcoxon signed-rank test]).

### Automatic ED and ES phase selection

**Fig. 2a:**
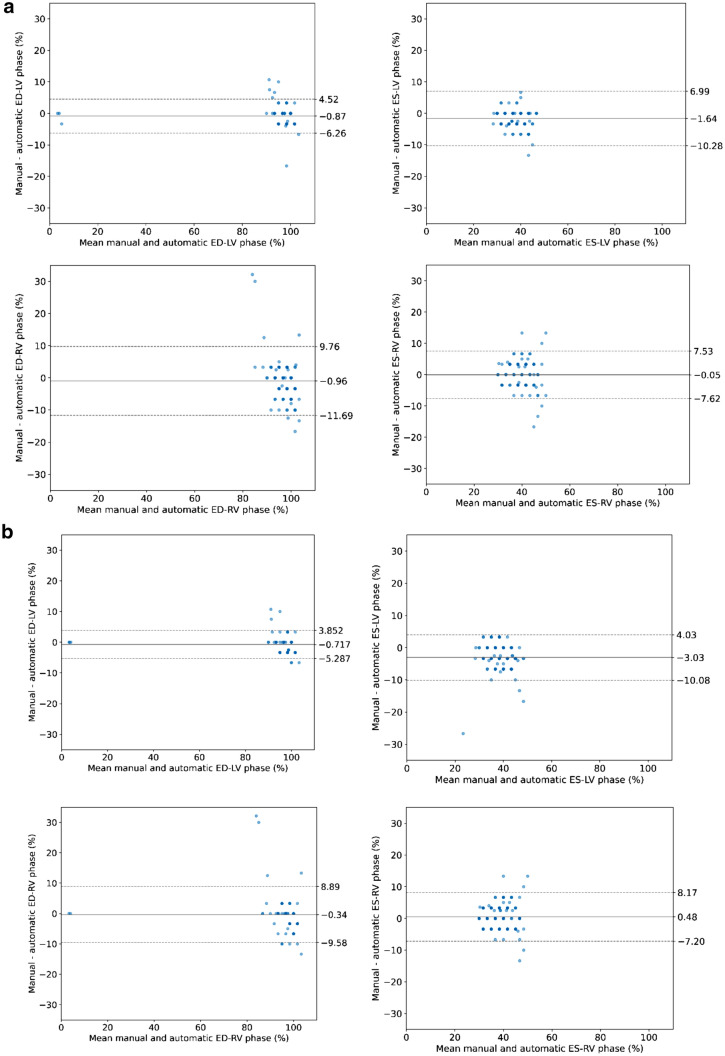
Bland–Altman plots with the agreement between the manually and automatically selected ED and ES phases for RV and LV, respectively, using *automatic* segmentations. **b** Bland–Altman plots with the agreement between the manually and automatically selected ED and ES phases for RV and LV, respectively, using *automatic*^*−basal*^ segmentations. Distance between automatically and manually selected phases is expressed as percentage of a complete cardiac cycle. Evaluation was performed for RV (top row) and LV (bottom row) separately. Higher opacity of colors correlates to higher density of data points. *Abbreviations: ED* = *end-diastolic; ES* = *end-systolic*

The Bland–Altman plots shown in Fig. 2a demonstrate the comparison between automatically identified cardiac phases using the automatic segmentations with the manually selected ED and ES phases. The bias [limits of agreement] were – 0.87 [ – 6.26, 4.52]% for the ED-LV phase and – 1.64 [ – 10.28, 6.99]% for the ES-LV phase, respectively, and – 0.96 [ – 11.69, 9.76]% for the ED-RV phase and –0.05 [-7.62, 7.53]% for the ES-RV phase, respectively. Figure 2b depicts the same comparison using the automatic^−basal^ segmentations to automatically determine the ED and ES phases. For this scenario the bias [limits of agreement] were – 0.72 [ – 5.29, 3.85]% for the ED-LV phase and – 3.03 [ – 10.08, 4.03]% for the ES-LV phase, respectively, and – 0.34 [ – 9.58, 8.89]% for the ED-RV and 0.48 [ – 7.20, 8.17]% for the ES-RV.

### Assessment of absolute CMR measurements

Automatically measured volumes (RV and LV EDV and ESV) are slightly underestimated compared to manually measured volumes (Supplementary Figs. 2 and 3). However, as shown in Table 4, the correlations of both RV and LV volumes were excellent (0.95–0.99, *p* < 0.001). For RV and LV EF and SV, automatic measurements seem to be slightly overestimated compared to manual measurements; nonetheless, correlations were excellent 0.82–0.89 for RV and good to excellent (0.78–0.93) for LV. After simulated manual correction of the basal slice, agreement between manual and automated measurements increased, as depicted in the Bland–Altman plots (Supplementary Figs. 2 and 3). This was also reflected in the Pearson correlation coefficient for both the volumetric (EDV, ESV) (*r* = 0.97–0.99, *p* < 0.001) as well as the functional (SV, EF) (*r* = 0.88–0.98, *p* < 0.001) CMR measurements.

**Table 4:** Correlation between manual and automatic measurements

	Mean absolute difference (vs. manual)	Correlation *r* (with manual)	Mean absolute difference (vs. manual) Basal corrected	Correlation *r* (with manual)
*Right ventricle*				
EF (%)	1.4 ± 4.7	0.82 (0.77–0.87)*	0.9 ± 3.9	0.88 (0.84–0.91)*
SV (ml)	– 2.0 ± 10.8	0.89 (0.84–0.91)*	0.7 ± 9.2	0.92 (0.90–0.94)*
EDV (ml)	– 9.9 ± 13.9	0.95 (0.94–0.97)*	– 5.5 ± 9.6	0.98 (0.97–0.98)*
ESV (ml)	– 7.9 ± 11.0	0.95 (0.93–0.96)*	– 4.8 ± 8.1	0.97 (0.96–0.98)*
*Left ventricle*				
EF (%)	2.4 ± 3.6	0.78 (0.71–0.84)*	1.4 ± 2.1	0.92 (0.89–0.94)*
SV (ml)	1.4 ± 7.3	0.93 (0.91–0.95)*	0.04 ± 4.2	0.98 (0.97–0.98)*
EDV (ml)	– 4.6 ± 6.1	0.99 (0.98–0.99)*	– 4.4 ± 4.1	0.99 (0.99–1.00)*
ESV (ml)	– 6.0 ± 6.4	0.95 (0.93–0.96)*	– 4.4 ± 4.6	0.97 (0.96–0.98)*

*EF* ejection fraction, *SV* stroke volume, *EDV* end-diastolic volume, *ESV* end-systolic volume

^*^p-value of correlation < 0.001

### Classification of ARVC TFC

**Fig. 3:**
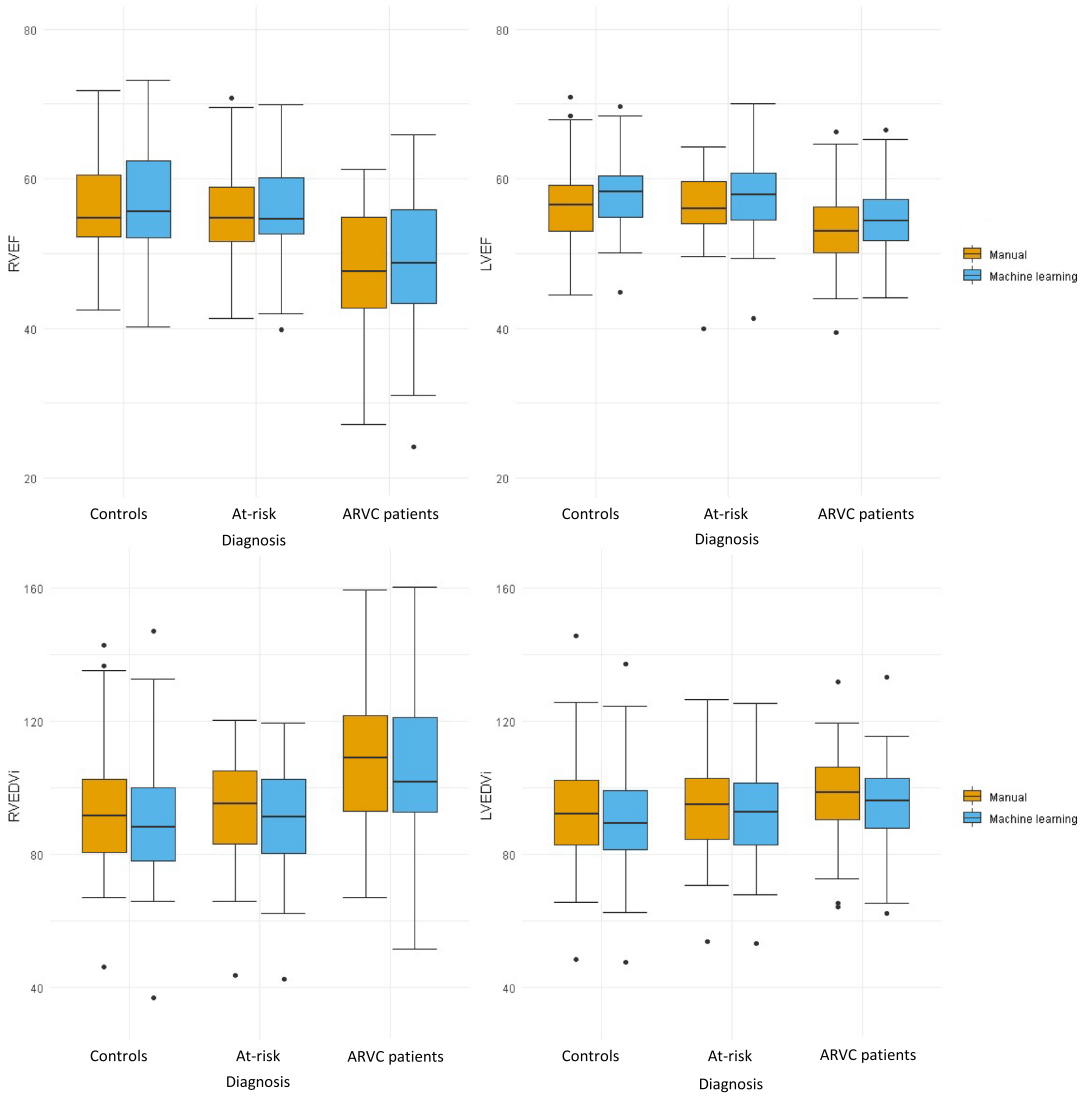
Boxplots depicting RV and LV function and dimension. CMR measurements are given for controls, at-risk family members and ARVC patients, stratified per method (manual [orange] vs. automatic [blue]). These data represent the automatic + basal correction data, see Supplementary Fig. 4 for the boxplots of the uncorrected automatic measurements. *Abbreviations: EDVI* = *end-diastolic volume index; EF* = *ejection fraction; LV* = *left ventricle; RV* = *right ventricle*

Since agreement between manual and automatic measurements was higher in the automatic^−basal^, we used these results for the further analysis. Supplementary Table 1 depicts the mean and standard deviation of the CMR measurements stratified per subgroup. The trends between the three subgroups (ARVC, at-risk family members and controls) were comparable between manual and automated measurements: ARVC patients had significantly reduced RVEF (*p* < 0.001) and LVEF (*p* = 0.002), as well as increased RVEDVI (*p* < 0.001), RVESVI (*p* < 0.001) and LVESVI (*p* < 0.013) compared to at-risk family members and controls. These trends between the subgroups were also observed in the boxplots of Fig. 3**.**

**Fig. 4:**
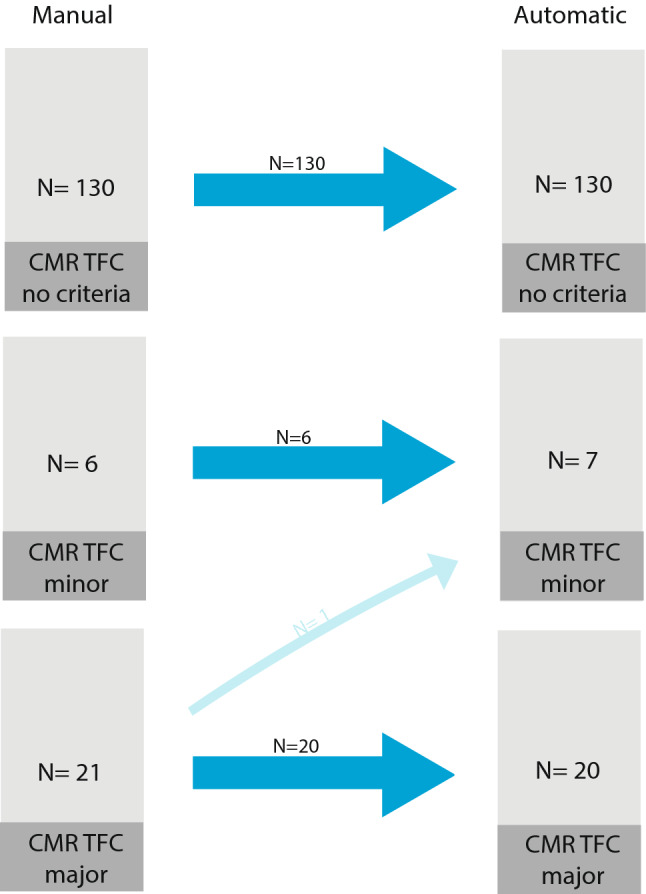
Classification of CMR criteria of TFC (no, minor and major) for manual and automatic + basal correction CMR measurements. The thick blue arrows indicate the matching subjects (between manual and automatic + basal correction), the thinner blue arrows indicate the number of patients that change CMR classification category when using automated measurements. See Supplementary Fig. 5 for uncorrected automatic measurements. *Abbreviations: CMR* = *cardiovascular magnetic resonance; N* = *number of subjects; TFC* = *Task Force Criteria*

We next compared CMR TFC classification using manual vs. automatic^−basal^ CMR measurements. All but one subject (156/157, 99%) were correctly classified, showing an agreement of κ 0.98 ± 0.02. As depicted in Fig. 4, subjects who classified as no (*n* = 130) or minor (*n* = 6) CMR TFC were correctly classified using the CMR measurements computed using the automatic segmentations obtained from the deep learning segmentation model. For major TFC, all but one subject were correctly classified; with one female subject being misclassified as minor CMR TFC. This classification discrepancy was based on a 5 ml/m^2^ difference in RVEDVI (102 ml/m^2^ using manual measurements and 97 ml/m^2^ using automatic measurements), whereby the cutoff for major CMR TFC is set at > 100 ml/m^2^ in women. The total TFC in this patient went from 5 to 4, which did not change the ARVC diagnosis. Sensitivity and specificity of minor and major CMR TFC for diagnosis of ARVC were comparable for manual (minor TFC 31% | 99% and major TFC 66% | 100%) and for automatic^−basal^ (minor TFC 35% | 100% and major TFC 65% | 100%, p = 0.32). CMR TFC classification using the uncorrected automatic measurements are depicted in Supplementary Fig. 5. This resulted in correct classification of 149/157 (95%) subjects.

## Discussion

In this study, we (i) evaluated our previously developed deep learning segmentation approach for RV and LV ventricular CMR assessment in patients suspected of ARVC and (ii) evaluated the clinical implication of this approach for classification of the CMR TFC in subjects suspected of ARVC. We demonstrated that CMR TFC classification using our automatic segmentation with limited manual correction in the most basal slice was comparable to classification using manual segmentation performed during clinical workup. Therefore, CMR TFC classification could potentially be performed using automatically measured CMR parameters with limited expert interaction.

### Previous studies

Recently studies [15, 23, 24] have shown that deep learning segmentation methods outperform traditional approaches such as those exploiting level set, graph-cuts, deformable models, cardiac atlases, and statistical models [25, 26]. Many current state-of-the-art deep learning biventricular segmentation algorithms have been evaluated on publicly available cine CMR data from the MICCAI 2017 ACDC [12]. The dataset contains CMR volumes from 150 patients distributed uniformly over normal cardiac function and four disease groups: dilated cardiomyopathy, hypertrophic cardiomyopathy, ischemic cardiomyopathy, and RV abnormality (RVEDVI greater than 110 mL/m^2^ for men, and greater than 100 mL/m^2^ for women, and/or a RVEF below 40%). The ACDC challenge showed that the largest segmentation inaccuracies were located in the most basal and apical slices of the short-axis [12], which is in line with our results presented in Table 3. Comparable results were obtained in the recently held Multi-Centre, Multi-Vendor and Multi-Disease Cardiac Segmentation (M&Ms) challenge [13]. Importantly, in contrast to the ACDC and M&Ms datasets, the clinical annotation protocol used in our study adheres to the guidelines of the SCMR [18]. Segmentation of the RV, especially in basal slices, is more challenging when following SCMR guidelines compared with the protocol used for the ACDC and M&Ms datasets. For example, in the SCMR guideline the outflow tract is included as part of the RV blood volume which challenges segmentation of the basal slices due to the unclear ventricular-atrial transition.

Researchers [27, 28] have also trained and evaluated deep learning CMR segmentation algorithms on the large-scale annotated dataset from the UK Biobank [29], reaching a performance comparable with human experts. The dataset contains short-axis cine CMR volumes of 5008 subjects. As the majority of the subjects are healthy, the dataset is considered relatively homogenous [29]. In the present work, we trained and evaluated a previously developed deep learning segmentation algorithm [16] on a real-life dataset with subjects suspected of ARVC who underwent CMR as part of their clinical evaluation. Compared to the previously mentioned datasets [12, 13, 29], our dataset contains substantially more subjects with RV complexity caused by ARVC due to possible aneurysms and wall thinning and contained CMR images acquired on different field strengths (1.5 and 3 Tesla), pulse sequences and imaging parameters. Hence, the current work demonstrates that by only correcting a single slice per volume, an existing state-of-the-art segmentation method [16] is sufficiently reliable to be applied to a relevant clinical problem. Furthermore, we are the first to compare classification of the CMR TFC of subjects suspected of ARVC using manually and automatically derived CMR measurements and showing that the deep learning segmentation algorithm we use performs well in this diverse clinical environment.

### Comparison to manual segmentation

We showed a good to excellent agreement of manual and automated CMR measurements, which significantly increased after simulated correction of the most basal slice of the RV and LV (automatic (*r* = 0.78–0.99, *p* < 0.001) and automatic^−basal^ (*r* = 0.88–0.99, *p* < 0.001) measurements). This was also reflected in the significant increase of the Dice coefficients and Hausdorff distance after basal correction (*p* < 0.001). This is in agreement with a recent study, showing an improvement of the agreement between automatic and manual segmentation when manually adjusting the most basal slice [30].

Large intra- and inter-observer variability is currently the greatest source of error when manually segmenting CMRs [8, 31] with more variability seen for the RV compared to LV due to the RV geometrical complexity [18]. Previously published inter-observer variability ranges from 2.6 – 10.5% [32, 33] for the LV and 6.2–14.1% [33, 34] for the RV. The largest variability between manual readers also appears in the apical and basal slices [14] presumably due to low tissue contrast ratios, hypertrabeculation and unclear ventricular-atrial transition of especially the RV. The variability in contouring of the basal slice is illustrated in Supplementary Fig. 1. The corresponding manual segmentations convey the difficulty to determine the anatomical boundaries of cardiac structures in these slices. We presumed that such variability also hampers performance of the automatic segmentation method. This limitation can be alleviated by increasing the size training set. To further improve performance of deep learning segmentation approaches, especially of basal and apical short-axis slices, future work could exploit anatomical information extracted from long-axis views (2-, 3-, 4-chamber views) e.g. valve landmarks and apical point [35, 36]. Furthermore, deep learning-based CMR segmentation methods would benefit from short-axis volumes with higher through-plane resolution (e.g. using super-resolution) [10, 37, 38]. This would make application of 3D segmentation approaches more feasible and hence, those models could potentially harness any inter-slice dependencies. Finally, using explicit topological prior information [39] for model optimization is a promising training approach to prevent automatic models from generating anatomically implausible segmentation.

### Clinical implementation of deep learning methods

Depending on the stage of disease, ARVC patients show a wide variety of ventricular changes that can be observed on CMR: ventricular wall motion abnormalities (e.g. aneurysms, akinesia, dyskinesia), wall thinning (due to fibrofatty replacement of the myocardium), increased trabeculations, dilatation and decreased functional measurements, that are especially present in the RV [2]. These challenges make ARVC eminently suitable to study the performance of machine learning algorithms on the RV. Previously published algorithms showed better agreement for LV than RV volumes [40]. Although limits of agreement were smaller for the LV compared to the RV, we showed comparable segmentation performance for RV and LV CMR measurements in this heterogenous study population. Furthermore, segmentation performance was comparable between structurally normal hearts and hearts affected by ARVC.

Importantly, we showed that calculation of ARVC TFC from automatically computed CMR parameters is feasible when combining automatic segmentation with correction of the most basal slice only. The diagnostic performance of the CMR TFC calculated using automatic segmentations (sensitivity 32–58%, specificity 99–100%) were comparable to manual measurements in this and previously published studies (sensitivity 13–69%, specificity 88–100%) [41, 42]. Although the correlation of manual and automatic measurements is high, the differences in CMR TFC classification without basal correction demonstrates that a fully automatic segmentation approach without human intervention is not yet reliable. However, the conducted experiments reveal that current state-of-the-art deep learning segmentation models can substantially reduce manual effort to semi-automatically segment cardiac structures in a heterogeneous dataset: manual segmentation time would be approximately 2 min instead of 25 min. Recently, Huellebrand et al.[43] proposed a human-in-the-loop approach that combines deep learning-based CMR segmentation and cardiac disease classification. The authors show that manual correction of automatic CMR segmentations by a clinical expert results in increased classification performance compared to a fully automatic segmentation approach. To identify volumes that contain segmentation failures the user can explore parallel coordinates plots that visualize CMR measurements along with cardiac shape and texture features. A similar approach was previously presented in Sander et al. [16] that combines automatic segmentation and assessment of segmentation uncertainty in CMR to automatically detect image regions containing local segmentation failures. Subsequently, detected regions are manually corrected by a clinical expert. Such a semi-automatic approach could lead to a large reduction in inter-observer variability. This is not only interesting for specialized tertiary ARVC centers, but even more for less experienced centers, since CMR misinterpretations are an important cause of over-diagnosis in ARVC and only 27% of people referred to a tertiary center with a suspected ARVC diagnosis finally meet diagnostic criteria for ARVC [44]. Our work shows that our previously developed deep learning segmentation method is able to fulfill a diagnostic purpose by simplifying accurate calculation of functional and volumetric measurements for the CMR TFC, showing opportunities to facilitate and improve individual patients health.

### Limitations

Although we automated the calculation of the dimensional and functional parameters, wall motion abnormalities are also part of the CMR TFC. This was evaluated visually by experienced cardiovascular radiologists in this work, but it is subject to inter-observer variation in less experienced readers. Due to anatomical challenges of the RV a fully automatic RV strain algorithm is not yet available. Future work should focus on automatic computation of RV strain and better automatic segmentation of the basal slice, which could contribute to full automatization and standardization of the CMR TFC.

Combining automatic segmentation with manual correction of the most basal slice, 99% of the CMR TFC were correctly classified, with misclassification of only one patient from major to minor CMR TFC. Moreover, one could argue that this latter classification falls within measurement error, and it did not change the diagnosis (total TFC score went from 5 to 4). Although the absolute differences in volumetric and functional parameters were small, due to the absolute cut-off values used for the CMR TFC, differences in classification can theoretically exist when the difference is as small as 1 ml/m^2^, and clinical interpretation of automatic measurements remains important. Notably, CMR is no gold standard for the diagnosis of ARVC, but rather part of the diagnostic process.

## Conclusions

Automatic deep learning-based CMR segmentation has the ability to provide a fast, standardized and reproducible method to measure RV and LV volumetric parameters on CMR. We demonstrate that the applied automated segmentations have a good agreement with manual segmentations. Furthermore, combining automatic segmentation with manual correction of the segmentation in the most basal slice results in accurate CMR TFC classification of subjects suspected of ARVC.

## Supplementary Information

Below is the link to the electronic supplementary material.

Supplementary file1 (DOCX 666 KB)

## Data Availability

All data generated or analyzed during this study are included in this published article [and its supplementary/additional information files].

## Abbreviations

ARVC: Arrhythmogenic right ventricular cardiomyopathy
CMR: Cardiovascular magnetic resonance
DRN: Dilated Residual Network
ED: End-diastole
EDV: End-diastolic volume
EDVI: End-diastolic volume index
EF: Ejection fraction
ES: End-systolic
ESV: End-systolic volume
ESVI: End-systolic volume index
LV: Left ventricle
RV: Right ventricle
SV: Stroke volume
TFC: Task force criteria
