# Supplementary material for: Towards automatic classification of cardiovascular magnetic resonance Task Force Criteria for diagnosis of arrhythmogenic right ventricular cardiomyopathy
Source: Clin Res Cardiol. 2022 Sep 6;112(3):363–78. doi: 10.1007/s00392-022-02088-x (PMC9998324; doi:10.1007/s00392-022-02088-x)
Supplement: Supplementary file 1 — Supplementary file1 (DOCX 666 KB) [file 392_2022_2088_MOESM1_ESM.docx]

**Additional files**

**Supplementary Table 1 CMR measurements stratified per subgroup**

*Uncorrected automated data*

|  | **ARVC patients**  **(n=37)** | **At-risk ARVC group (n=66)** | **Control group**  **(n=54)** | **p-value** |
| --- | --- | --- | --- | --- |
| **Manual measurements** | | | | |
| *Right ventricle* | | | |  |
| EF | 47.1 ± 9.0**‡ | 55.5 ± 5.9 | 56.2 ± 6.1 | <0.001 |
| SV | 99.0 ± 15.9‡ | 92.7 ± 18.3 | 99.5 ± 23.4 | 0.116 |
| EDV | 218.1 ± 53.2**‡ | 168.6 ± 34.9 | 178.1 ± 41.3 | <0.001 |
| EDVI | 111.6 ± 25.4**‡ | 93.7 ± 14.8 | 92.9 ± 18.5 | <0.001 |
| ESV | 119.1 ± 45.8**‡ | 76.0 ± 20.8 | 78.6 ± 22.6 | <0.001 |
| ESVI | 60.8 ± 22.7**‡ | 42.1 ± 9.9 | 41.1 ± 11.2 | <0.001 |
|  |  |  |  |  |
| *Left ventricle* | | | |  |
| EF | 53.3 ± 5.7**‡ | 56.4 ± 4.4 | 56.7 ± 5.3 | 0.003 |
| SV | 101.0 ± 18.1 | 94.6 ± 18.3 | 101.3 ± 23.1 | 0.133 |
| EDV | 190.5 ± 33.4‡ | 168.6 ± 33.6 | 179.0 ± 38.3 | 0.011 |
| EDVI | 97.5 ± 14.3 | 93.6 ± 12.8 | 93.2 ± 16.0 | 0.322 |
| ESV | 89.6 ± 20.7**‡ | 74.0 ± 18.3 | 77.7 ± 19.0 | 0.001 |
| ESVI | 45.6 ± 8.7**‡ | 41.0 ± 7.9 | 40.5 ± 8.9 | 0.010 |
|  |  |  |  |  |
| **Uncorrected automatic measurements** | | | | |
| *Right ventricle* | | | | |
| EF | 49.2 ± 9.0**‡ | 56.9 ± 5.8 | 57.4 ±7.7 | <0.001 |
| SV | 98.5 ± 21.8 | 90.2 ± 20.5 | 97.0 ± 26.6 | 0.139 |
| EDV | 204.7 ± 51.0**‡ | 159.9 ± 35.3 | 169.2 ± 41.9 | <0.001 |
| EDVI | 104.6 ± 24.0**‡ | 88.7 ± 15.1 | 88.0 ± 18.1 | <0.001 |
| ESV | 106.2 ± 39.5**‡ | 69.7 ± 19.4 | 72.3 ± 22.7 | <0.001 |
| ESVI | 54.3 ± 19.5**‡ | 38.6 ± 8.9 | 37.7 ± 11.1 | <0.001 |
|  |  |  |  |  |
| *Left ventricle* | | | | |
| EF | 55.6 ± 5.7**‡ | 58.7 ± 5.6 | 59.3 ± 5.3 | 0.005 |
| SV | 102.8 ± 17.9 | 95.8 ± 17.0 | 102.6 ± 22.6 | 0.091 |
| EDV | 186.2 ± 32.8‡ | 164.6 ± 32.3 | 173.5 ± 36.9 | 0.010 |
| EDVI | 95.3 ± 14.3 | 91.4± 12.7 | 90.4 ± 15.4 | 0.246 |
| ESV | 83.3 ± 20.0**‡ | 68.8 ± 19.2 | 70.9 ±18.3 | 0.001 |
| ESVI | 42.5 ± 9.0**† | 38.1 ± 8.7 | 36.9 ± 8.5 | 0.009 |

Significant difference 0.01-0.05 (*) or (**<0.01) between control and ARVC patients

Significant difference 0.01-0.05 (†) or (‡<0.01) between at-risk and ARVC patients

*Corrected automated measurements with simulated correction of the most basal slice*

|  | **ARVC patients**  **(n=37)** | **At-risk ARVC group (n=66)** | **Control group**  **(n=54)** | **p-value** |
| --- | --- | --- | --- | --- |
| **Manual measurements** | | | | |
| *Right ventricle* | | | |  |
| EF | 47.1 ± 9.0**‡ | 55.5 ± 5.9 | 56.2 ± 6.1 | <0.001 |
| SV | 99.0 ± 15.9‡ | 92.7 ± 18.3 | 99.5 ± 23.4 | 0.116 |
| EDV | 218.1 ± 53.2**‡ | 168.6 ± 34.9 | 178.1 ± 41.3 | <0.001 |
| EDVI | 111.6 ± 25.4**‡ | 93.7 ± 14.8 | 92.9 ± 18.5 | <0.001 |
| ESV | 119.1 ± 45.8**‡ | 76.0 ± 20.8 | 78.6 ± 22.6 | <0.001 |
| ESVI | 60.8 ± 22.7**‡ | 42.1 ± 9.9 | 41.1 ± 11.2 | <0.001 |
|  |  |  |  |  |
| *Left ventricle* | | | |  |
| EF | 53.3 ± 5.7**‡ | 56.4 ± 4.4 | 56.7 ± 5.3 | 0.003 |
| SV | 101.0 ± 18.1 | 94.6 ± 18.3 | 101.3 ± 23.1 | 0.133 |
| EDV | 190.5 ± 33.4‡ | 168.6 ± 33.6 | 179.0 ± 38.3 | 0.011 |
| EDVI | 97.5 ± 14.3 | 93.6 ± 12.8 | 93.2 ± 16.0 | 0.322 |
| ESV | 89.6 ± 20.7**‡ | 74.0 ± 18.3 | 77.7 ± 19.0 | 0.001 |
| ESVI | 45.6 ± 8.7**‡ | 41.0 ± 7.9 | 40.5 ± 8.9 | 0.010 |
|  |  |  |  |  |
| **Corrected automatic measurements** | | | | |
| *Right ventricle* | | | | |
| EF | 48.3 ± 9.6**‡ | 56.1 ± 6.0 | 57.2 ±7.4 | <0.001 |
| SV | 98.7 ± 20.7 | 91.6 ± 21.0 | 99.0 ± 27.3 | 0.159 |
| EDV | 210.1 ± 50.5**‡ | 164.1 ± 35.5 | 173.2 ± 42.3 | <0.001 |
| EDVI | 107.4± 23.8**‡ | 91.1 ± 14.9 | 90.1 ± 18.5 | <0.001 |
| ESV | 111.4 ± 41.8**‡ | 72.5 ± 19.3 | 74.1 ± 22.2 | <0.001 |
| ESVI | 56.9 ± 20.7**‡ | 40.2 ± 9.0 | 38.8 ± 11.1 | <0.001 |
|  |  |  |  |  |
| *Left ventricle* | | | | |
| EF | 54.6 ± 5.2**‡ | 57.8 ± 4.9 | 58.1 ± 5.2 | 0.003 |
| SV | 100.7 ± 18.0 | 94.9 ± 18.1 | 101.3 ± 23.7 | 0.173 |
| EDV | 185.4 ± 33.4‡ | 165.1 ± 33.0 | 174.2± 37.5 | 0.018 |
| EDVI | 94.8 ± 14.4 | 91.7 ± 12.9 | 90.7 ± 15.5 | 0.376 |
| ESV | 84.7 ± 19.5**‡ | 70.2 ± 18.2 | 72.9 ±17.6 | 0.001 |
| ESVI | 43.2 ± 8.5**† | 38.9 ± 8.1 | 38.0 ± 8.4 | 0.011 |

Significant difference 0.01-0.05 (*) or (**<0.01) between control and ARVC patients

Significant difference 0.01-0.05 (†) or (‡<0.01) between at-risk and ARVC patients

*Abbreviations as in manuscript*

| 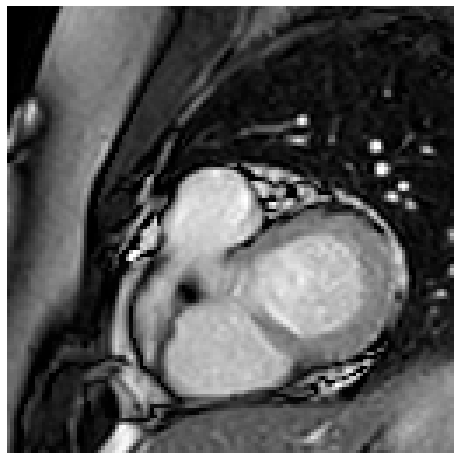 | 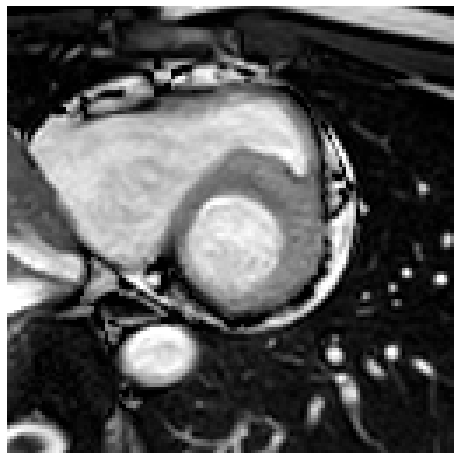 | 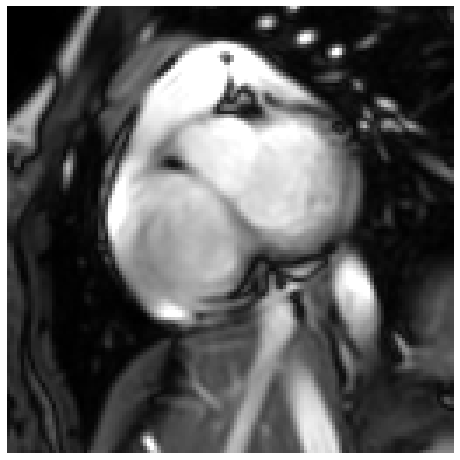 | 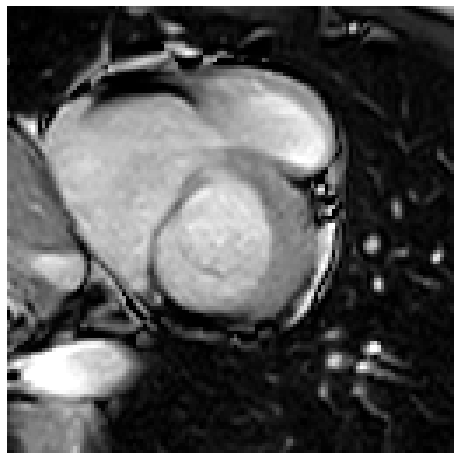 |
| --- | --- | --- | --- |
| 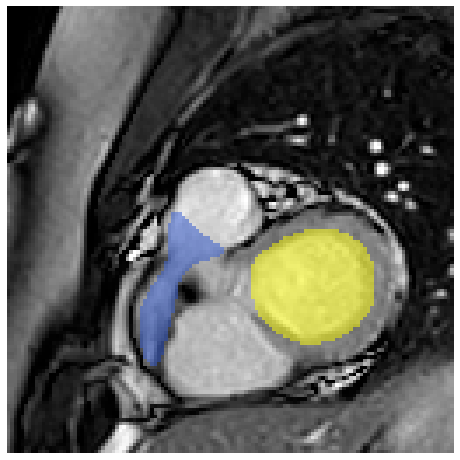 | 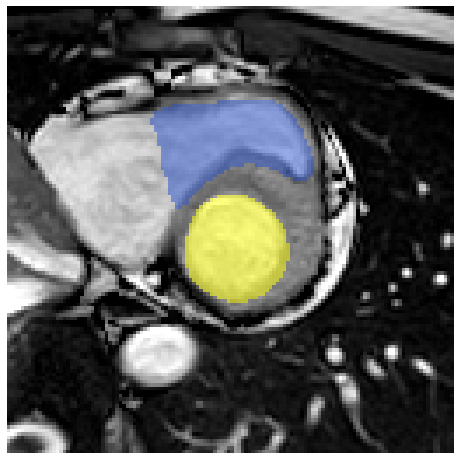 | 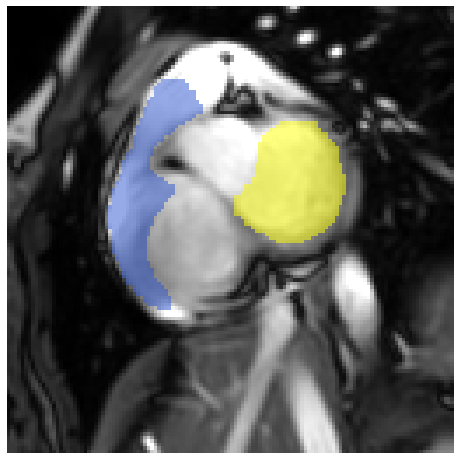 | 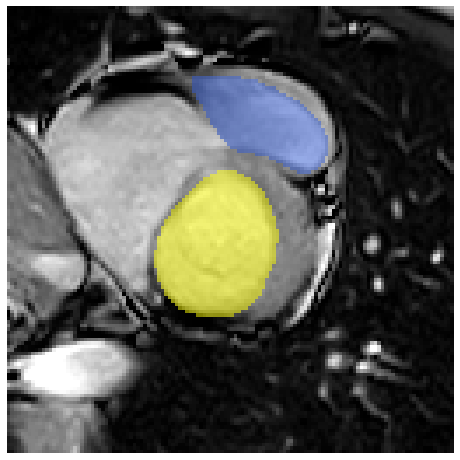 |

**Supplementary Figure 1. Examples illustrating RV shape variability in manual reference segmentations for basal slices.**

Shown are original CMR without (top row) and with (bottom row) manual reference segmentations of the left (yellow) and right (blue) ventricle.

| Without basal correction | 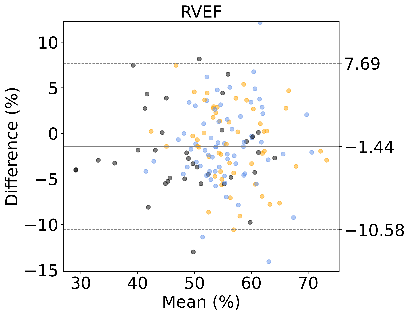 | 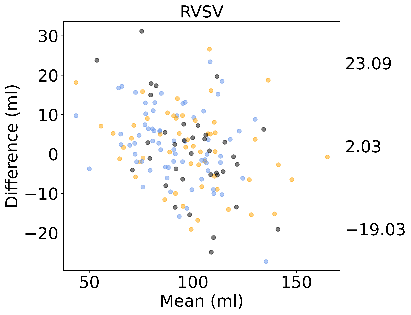 | 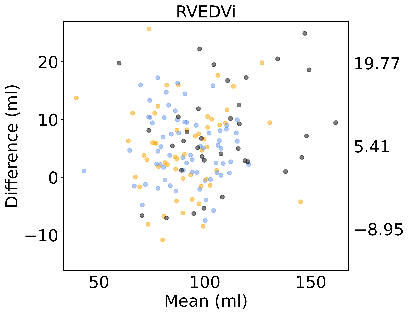 | 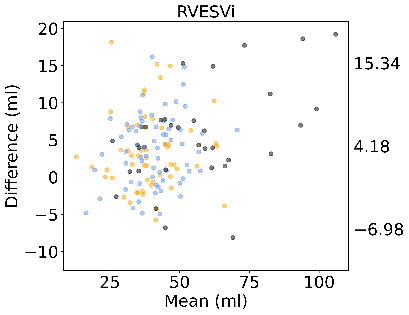 |
| --- | --- | --- | --- | --- |
| With basal correction | 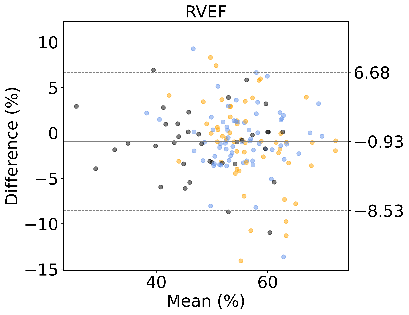 | 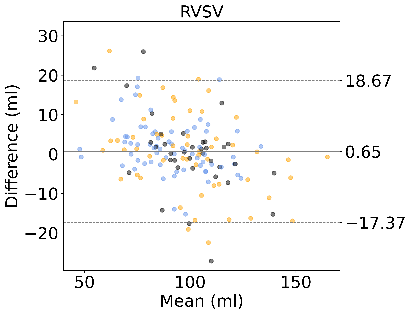 | 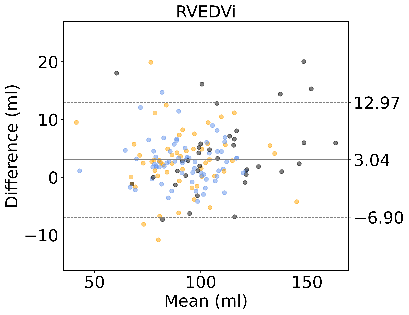 | 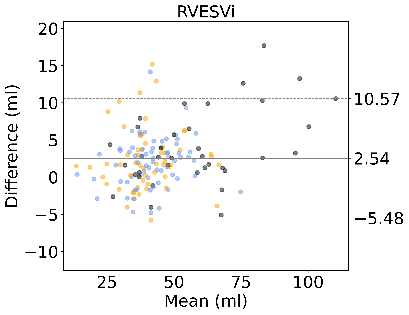 |

**Supplementary Figure 2. Bland-Altman plots of right ventricular CMR measurements**

Absolute agreement between: (top row) manuals vs. uncorrected automatic CMR measurements; and (bottom row) manuals vs. automatic+ basal correction CMR measurements. Data points are stratified by disease classification 1) ARVC patients (in black); 2) at-risk family members (in blue) and 3) control subjects (in orange).

*Abbreviations as in manuscript.*

| Without basal correction | 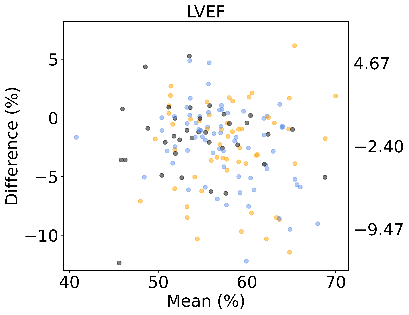 | 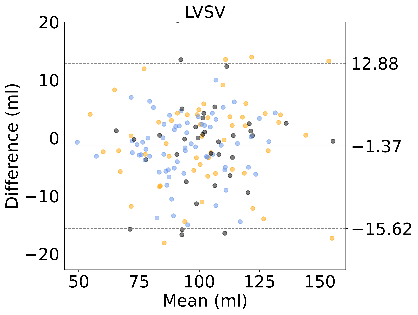 | 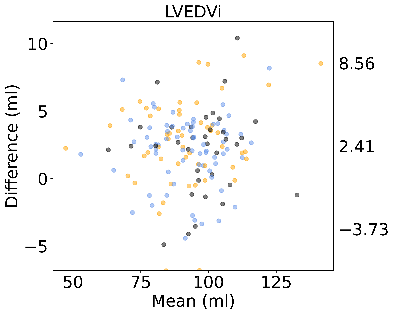 | 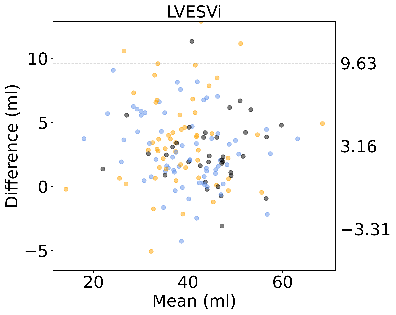 |
| --- | --- | --- | --- | --- |
| With basal correction | 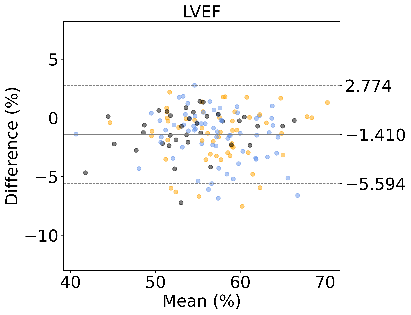 | 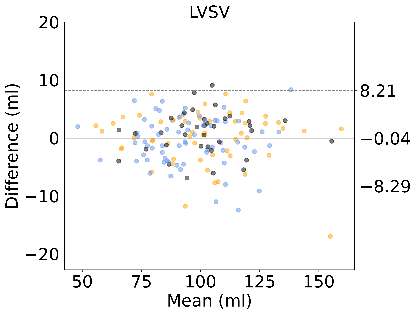 | 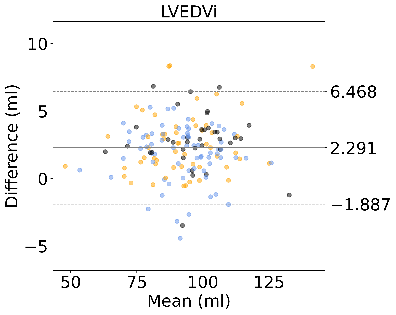 | 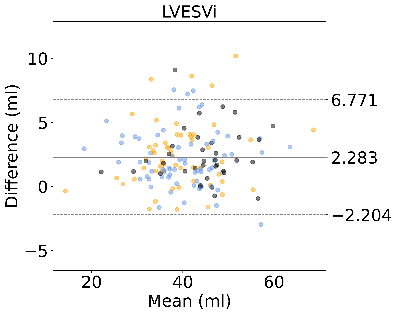 |

**Supplementary Figure 3. Bland-Altman plots of left ventricular CMR measurements**

Absolute agreement between: (top row) manuals vs. uncorrected automatic CMR measurements; and (bottom row) manuals vs. automatic + basal correction CMR measurements. Data points are stratified by disease classification 1) ARVC patients (in black); 2) at-risk family members (in blue) and 3) control subjects (in orange).

*Abbreviations as in manuscript.*


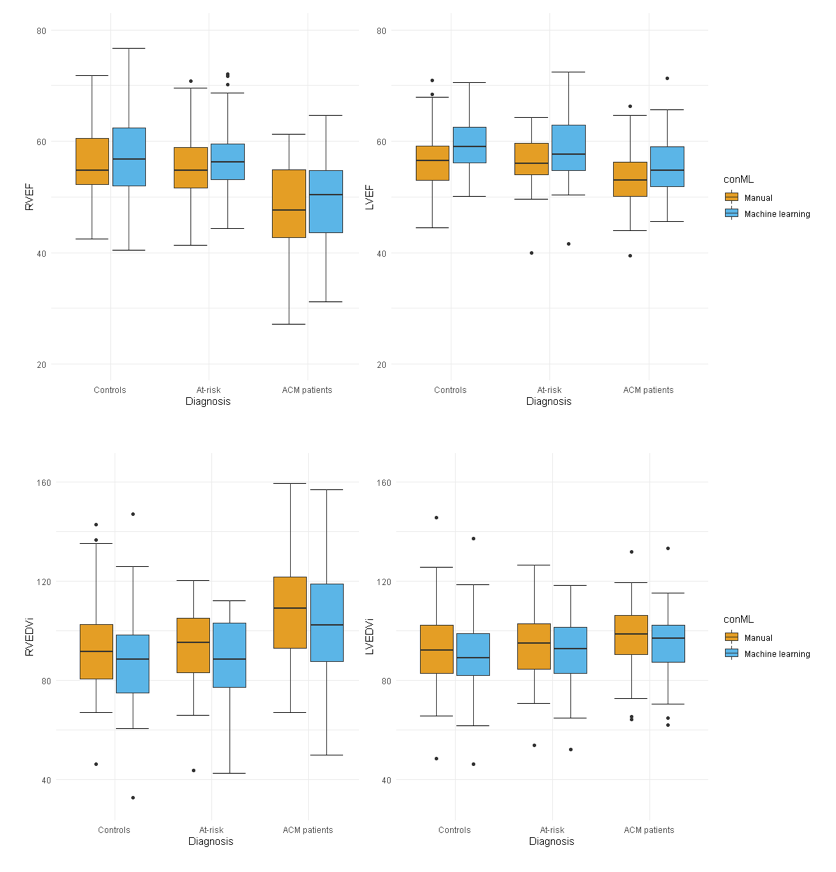


**Supplementary Figure 4. Boxplots depicting RV and LV function and dimension**.

This data represents the basal uncorrected automatic data. CMR measurements are given for controls, at-risk family members and ARVC patients, stratified per method (manual [orange] vs. automatic [blue]).

*Abbreviations: EDVI= end-diastolic volume index; EF= ejection fraction; LV= left ventricle; RV= right ventricle*

**Supplementary Figure 5. Classification of CMR criteria of TFC (no, minor and major) for manual and uncorrected automatic CMR measurements.** The thick blue arrows indicate the matching subjects (between manual and automatic), the thinner blue arrows indicate the number of patients that change CMR classification category when using automated measurements. Cohen’s Kappa between manual and automatic measurements was κ 0.82±0.05. Minor TFC, sensitivity 32% and specificity 100%; major TFC, sensitivity 58% and specificity 99%).

*Abbreviations: CMR= cardiovascular magnetic resonance; N= number of subjects; TFC= task force criteria*
